# Emerging Healthcare Trends in Prosthetic Treatment of Hand Osteoarthritis

**DOI:** 10.3390/jcm14020573

**Published:** 2025-01-17

**Authors:** Andreas M. Siegmund, Marc Ruewe, Dominik Szymski, Rafael Loucas, Dmytro Oliinyk, Andrea Pagani, Cassandra Ecklmaier, Sebastian Geis, Alexandra M. Anker, Lukas Prantl, Silvan M. Klein

**Affiliations:** 1 Department of Plastic, Hand, and Reconstructive Surgery, University Hospital Regensburg, 93053 Regensburg, Germany; andreas.siegmund@ukr.de (A.M.S.); marc.ruewe@ukr.de (M.R.); rafael.loucas@ukr.de (R.L.); dmytro.oliinyk@ukr.de (D.O.); andrea.pagani@ukr.de (A.P.); cassandra.ecklmaier@stud.uni-regenburg.de (C.E.); sebastian.geis@ukr.de (S.G.); alexandra.anker@ukr.de (A.M.A.); lukas.prantl@ukr.de (L.P.); 2 Department of Trauma Surgery, University Hospital Regensburg, 93053 Regensburg, Germany; dominik.szymski@ukr.de

**Keywords:** prostheses, osteoarthrosis, arthrosis, finger joint, carpometacarpal joints, thumb, arthroplasty, replacement, finger, prosthesis implantation, revision surgery, joint revision, osteoarthritis, metacarpophalangeal joints, finger interphalangeal joints

## Abstract

**Background**: For many years, advancements in hand joint replacement (JR) were relatively minor compared to those for large joints. However, the caution previously exercised due to high complication rates is gradually being replaced by the expanding use of JR therapies for small joints in the hand. Despite this progress, there is a lack of comprehensive data on the outcomes of hand JR and on the optimal infrastructure required to meet the growing demand for these therapies. **Methods**: This study examined trends and revision rates of JR for thumb carpometacarpal (CMC-1) and finger (MCP and PIP) joints in both inpatient and outpatient settings in Germany. Data from the Federal Statistical Office of Germany (Destatis) and the Central Institute for Statutory Health Insurance Physicians (ZI) were analyzed, focusing on the incidence, demographics, and outcomes of these procedures. **Results**: This study found a substantial national increase in prosthetic treatments specifically for CMC-1, with a 2.18-fold rise in the outpatient sector compared to a 1.65-fold increase in inpatient treatments. Despite this shift, 83.7% of JR procedures were still performed in an inpatient setting. **Conclusions**: The overall complication rates appear to be declining, suggesting that while the management of these procedures is shifting towards outpatient care, the quality remains stable.

## 1. Introduction

Osteoarthritis (OA) is the leading cause of disability among joint diseases [1]. More than 527 million people worldwide are currently affected by OA, and its prevalence is projected to rise further as populations continue to age [2]. OA affects articular cartilage, which is a highly specialized tissue that allows for smooth, pain-free, and almost frictionless movement of the joints [3]. However, the highly limited regenerative capacity of articular cartilage following damage from trauma or degeneration has prompted the development of numerous therapeutic strategies, ranging from cartilage repair techniques to full joint replacement (JR) [1,2,4]. Despite the advancement of various regenerative approaches, JR often remains the ultimate solution for maintaining a painless range of motion [1,5]. Of note, hand joints are the second most affected sites in OA, following the knee joint [2]. Moreover, OA in small joints, including the thumb carpometacarpal (CMC-1), proximal interphalangeal (PIP), and metacarpophalangeal (MCP) joints, can severely impair hand function. If left untreated, OA-induced pain and reduced grip strength ultimately lead to a diminished quality of life for those affected [6].

Whereas early-stage OA is typically managed conservatively [7], surgical options are considered for more advanced conditions [8]. In the past, the historical absence of bona fide implants has led to numerous competing surgical techniques, all aimed at avoiding JR while preserving a more or less optimal range of motion [9,10,11]. Notably, especially compared to large joints such as the knee or hip, advancements in hand JR have been relatively minor with unacceptable complication rates reaching up to 35% until recently [12].

To date, proven concepts from large joint implants have successfully contributed to the evolution of small joint prostheses, resulting in highly predictable clinical outcomes and, hence, has made these implants the current gold standard for PIP and MCP OA care [9]. Moreover, the latest advances in joint implants are not restricted to PIP and MCP joints [9]. In the past decade, the latest versions of CMC-1 prostheses appear to have finally overcome the issues of the high failure rates of previous implant generations [13].

In fact, recent data suggest that the functional outcomes of CMC-1 JR might be superior when compared to other established surgical techniques [14]. Consequently, according to manufacturers, the total number of CMC-1 prostheses implanted in Europe alone reached 52,000 between 2009 and 2022 [13].

These numbers indicate an essential paradigm shift towards JR in the management of OA in hand surgery. Nevertheless, novel therapeutic approaches inevitably face the challenge of cost-effective implementation within chronically overburdened healthcare systems—a difficult lesson learned from the financial strain of past substantial expansions in hip and knee JR care [15].

However, the lack of comprehensive data on the outcomes of hand joint prosthetics currently stifles any objective discussion. Therefore, this study aims to address this gap in the knowledge by thoroughly comparing large datasets from both inpatient as well as outpatient settings, contributing to a rational discussion on the optimal infrastructure to meet the rapidly growing demand for hand JR.

## 2. Materials and Methods

This study retrospectively examined patients who received endoprosthetic procedures performed on the thumb carpometacarpal (CMC-1) joint and finger (MCP and PIP) joints in Germany from January 2012 to December 2023. The analysis included surgical codes (OPS-Codes) describing prosthetic treatments of the CMC-1 and finger joints. Additionally, revision surgeries for CMC and finger prostheses were also analyzed (Table 1). The inpatient data, sourced from the Federal Statistical Office of Germany (Destatis, Wiesbaden), were compared to outpatient data sourced from the Central Institute for Statutory Health Insurance Physicians in Germany (ZI, Berlin). The ZI data basis includes nationwide outpatient billing data according to §295 SGB V for the years 2013 to 2022. It includes GKV (statutory health insurance) patients who had contact with an outpatient doctor at least once in the respective year. The data for this analysis were retrieved from Destatis on 30 May 2024 and from ZI on 25 May 2024, and all the data were anonymized for privacy. The data used in this study were provided in 20-year age brackets, which guided our approach to age-based analyses. Since the study utilized anonymized data from a centralized administrative database, there was no need for informed consent or institutional review board (IRB) approval.

We analyzed the inpatient and outpatient data on patients, focusing on the total case numbers, incidence, and demographic details (age). The data are presented as absolute and relative frequencies. The incidence (IR) and age-adjusted (AAIR) incidence rates were calculated based on Germany’s historical population provided by Destatis. With regard to finger prostheses, the AAIR focused on two 20-year age brackets (i.e., age groups 40–59 and 60–79), as these two specific age groups accounted for over 90% of the treated population. Moreover, the very low number of outpatient treatments in the other 20-year brackets (i.e., age groups 0–19, 20–39, >80) did not allow for an adequate comparison with the inpatient treatments. The incidence rate (IRR) and confidence interval (CI) for the prosthetic procedures were calculated. The population of each of the 16 German federal states was analyzed by the year of birth the years from 2013 and 2022, with data recorded as of 31 December each year. The chi-squared test was employed to compare variables, with a significance threshold set at *p* ≤ 0.05. The OR and 95% CI for in-hospital mortality were calculated. Statistical analyses and figure design were performed using GraphPad Prism v10.2.2 (GraphPad Software, Boston, MA, USA).

## 3. Results

Between 2012 and 2023, a total of 22,752 procedures were performed for endoprosthetic care were performed. When prostheses were implanted, they were more frequently located in the finger joints (55%, 10,719/19,501). The great majority of these procedures, including the initial treatments and revision surgeries for both the CMC-1 and finger joints, were performed in an inpatient setting (Table 2).

**Figure 1:**
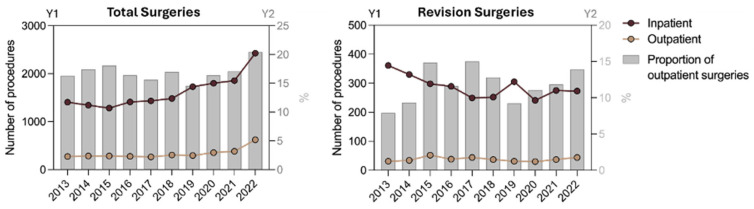
The diagrams illustrate the annual trend in the number of cases of joint implantation (CMC-1 and finger joint prostheses combined, (**left**) and revision surgery (**right**)). Absolute numbers per year (Y1) are indicated for inpatient and outpatient procedures. The proportion of outpatient operations is shown as a percentage bar (Y2).

Over the decade studied, both the outpatient and inpatient sectors showed an overall increase in prosthetic treatments. The share of outpatient joint replacement increased from 16.3% to 20.4%. Regarding the revision procedures performed, there was an overall decrease during this period while the share of revisions performed in the outpatient increased from 7.9% to 13.9% (Figure 1).

**Figure 2:**
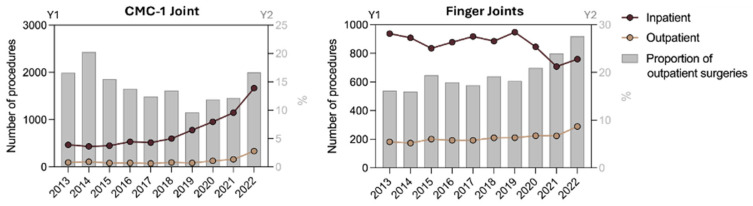
The diagrams illustrate the annual trend in the number of cases of joint replacement for CMC-1 (**left**) and finger joint prostheses (**right**). Absolute numbers per year (Y1) are indicated for inpatient and outpatient procedures. The proportion of outpatient operations is shown as a percentage bar (Y2).

In total, 8782 CMC-1 and 10,719 finger joint prostheses were implanted, with 14.1% and 19.6%, respectively, in the outpatient sector. For CMC-1 joint replacements, there was a strong increase in inpatient and outpatient cases. Inpatient finger joint replacements decreased over the years studied while the outpatient sector rose. The proportion of outpatient care for CMC-1 joint prostheses fluctuated greatly while the share of outpatient finger joint replacement grew steadily (Figure 2).

Looking at the incidence rate (IR) and the incidence rate ratio (IRR), one sees an overall increase in joint replacements while the number of CMC-1 replacements increased greatly, and finger joint replacements even showed a slight decrease. For the CMC-1 joint replacements, the outpatient sector and inpatient sector experienced a substantial rise (IRR 3.44 vs. IRR 3.42). Outpatient finger joint replacements demonstrated an increase as well while the inpatient sector greatly decreased (IRR 1.53 vs. IRR 0.77). Revisions decreased overall in the period under review, with a trend towards more revisions being carried out in the outpatient sector (Table 3).

**Figure 3:**
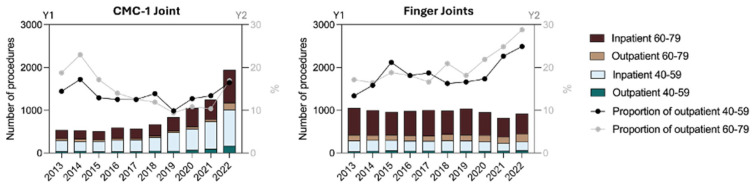
The trend in inpatient and outpatient care for age groups 40–79. Absolute numbers per year (Y1) and the share of outpatient care of total care in percentage per year (Y2).

In most cases (90.7% of outpatient and 90.9% of inpatient care), the patient undergoing finger joint replacement operations were between 40 and 80 years old. The analysis of the data by age groups showed that the number of outpatient cases increased, with a more pronounced increase for the 40–59 age group (IRR 1.82, 95% CI 1.23 to 2.71; *p* = 0.0026). Both age groups experienced significant increases in the number of CMC-1 replacements from 2013 to 2022. This increase is evident in both the outpatient and inpatient settings. While both settings saw significant increases, the outpatient procedures in the 40–59 age group saw the highest relative increase (IRR of 4.21, 95% CI 3.01 to 5.88; *p* < 0.001) (Figure 3, Table 4).

## 4. Discussion

### Increase in Procedures and Shift Towards Outpatient Care

The data analyzed in this study revealed a substantial increase in prosthetic treatments exclusively for the CMC-1 joint, while replacements of finger joints declined (Figure 2, Table 3). Most likely, this trend can be attributed to both medical and economic factors. Decreasing complication rates [12] and increasing patient satisfaction with prosthetic care have led hand surgeons [16] worldwide to expand indications for JR over the past two decades [17]. Furthermore, original legal barriers to outpatient procedures were removed in Germany to conserve national inpatient resources and limit discretionary healthcare spending [18]. Despite the increasing shift towards outpatient care, our study highlights a substantial difference between the number of procedures performed in the inpatient sector (83.7%) and the outpatient sector (16.3%) among all JR procedures.

Although the national shift from inpatient to outpatient settings is becoming increasingly evident, it is important to emphasize that only 14.1% of the 8782 CMC-1 joint prostheses have been implanted in the outpatient sector to date. Whether this imbalance is predominantly driven by the practice of defensive medicine, concerns about litigation, or simply a lack of available infrastructure remains unclear at this point.

Despite the COVID-19 pandemic acting as a catalyst for the transition from inpatient to outpatient care, there is still hesitation in many areas regarding the expansion of outpatient care structures [19]. Indeed, in 2020, the German Society for Hand Surgery published a consensus statement explicitly recommending prosthetic treatment for the CMC-1 joint and finger joints in an inpatient setting [20]. Essentially, this underlines the overall conservative attitude towards outpatient care in Germany in comparison to international standards [21]. In contrast, Nisar et al. compared inpatient and outpatient hand JR and found that outpatient JR was more cost-efficient while being equally as effective as procedures performed in an inpatient setting [22].

However, the clash between the moral implications of medical care as a social good and the laws of the market economy is becoming increasingly evident in public discussions on optimal care, which certainly extends beyond hand surgery [23]. Still, an essential prerequisite for a constructive debate on balancing economics and quality of care is recognizing healthcare research as a powerful tool to optimize patient management. In this regard, early versions of implants for JR in the hand suffered from high complication rates, which frequently necessitated surgical revision [12].

**Figure 4:**
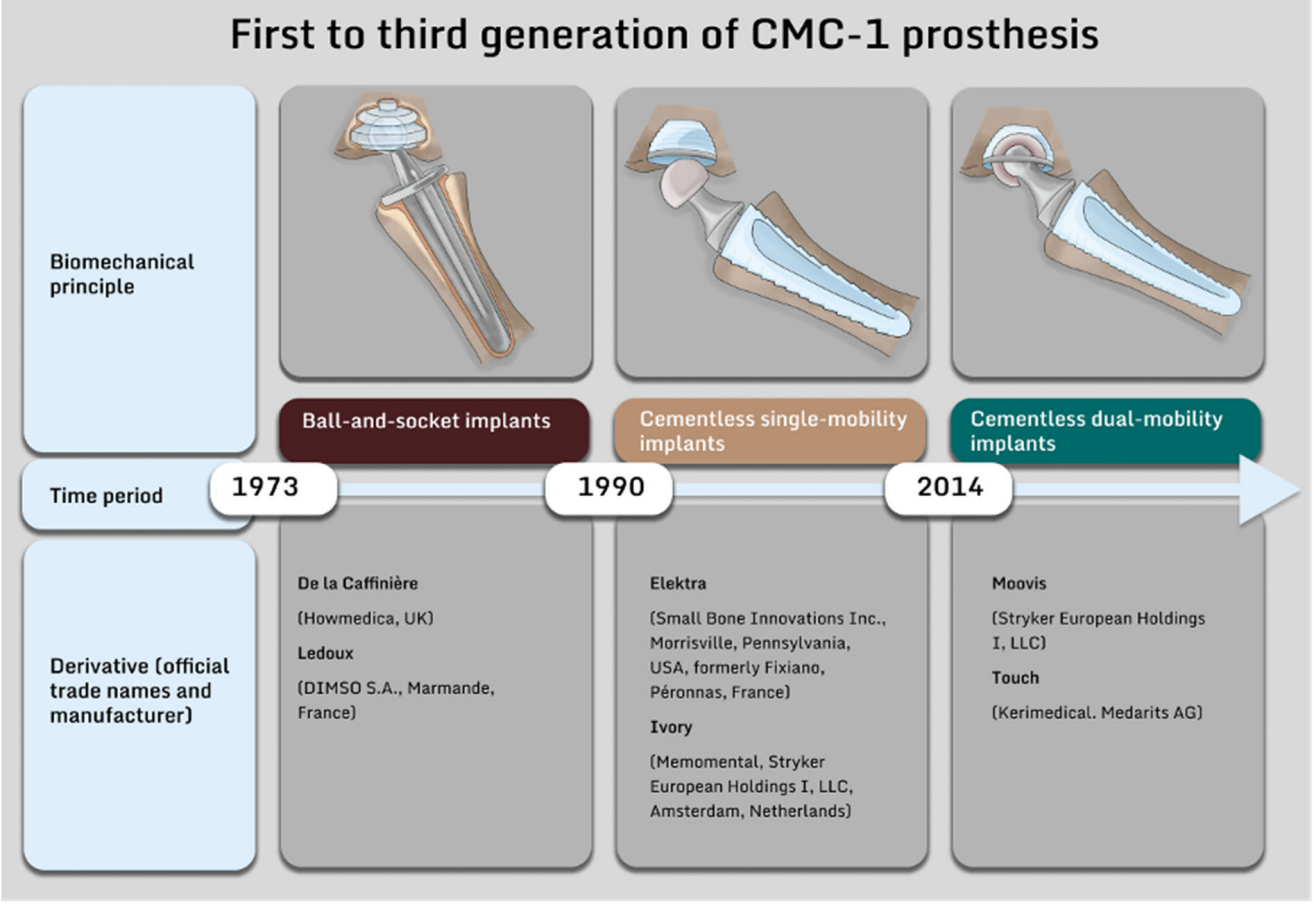
The first generation of total endoprostheses, based on the ball-and-socket principle, yielded high loosening rates in both cups and stems [25]. A second, cementless generation improved implant longevity through enhanced osseointegration, though its rigid center of rotation increased the risk of dislocation [26]. This issue appears largely resolved in recent implant designs, which incorporate the dual mobility concept [27,28,29,30].

However, our study revealed an overall decreasing trend for revision surgery. The decline in revision rates may be attributed to substantial improvements in the design of third-generation CMC-1 prostheses, which have minimized the loosening and dislocation tendencies seen in earlier implant iterations (Figure 4). The trend toward more outpatient procedures also becomes evident in revision cases. Moreover, the IRR is slightly positive in the outpatient sector and is clearly declining in the inpatient sector (Table 3).

Notably, a similar analysis of national data in the U.S. reported an average 35% complication rate [24]. Since the U.S. data were collected between 2009 and 2016, we suspect that the higher complication rate may be attributed to the less refined implant technology used during that period (Figure 4). Essentially, modern implant designs, combined with increased expertise among practitioners, have presumably overcome the growing pains of early hand joint prosthetic surgery.

Another aspect could be the concurrent advancements in medical treatments for rheumatoid diseases, enabling JR earlier in the disease stage due to the more potent suppression of synovitis along with fewer side effects [31]. Hence, in rheumatoid conditions, a well-timed JR with improved soft tissue conditions and preserved ligament support promises better overall outcomes [31]. Along with an increased confidence in implant technology among providers, this may have driven the shift from a fallback therapy to an accepted gold standard [9]. Moreover, this more liberal selection of JR as a treatment option for younger patients may have positively contributed to stabilizing revision rates due to the improved baseline health conditions of JR candidates.

Nevertheless, this study is limited by its retrospective nature and reliance on administrative data. Undisputedly, these large datasets benefit from their unfiltered and robust validity. However, this great value of large datasets comes at the cost of data noise, which may obscure certain nuances. This limitation inherently raises the risk of biased conclusions, as certain underlying factors may not have been sufficiently accounted for in the analysis. Although the primary focus of this study was to examine hand JR data from a macro perspective, including its epidemiological dimensions and infrastructure-related quality of care, it is important to emphasize that the data did not allow us to assess clinical outcomes such as functional aspects or patient-reported outcomes, which are, without a doubt, crucial for an evaluation of the clinical effectiveness. These metrics are critical for assessing the effectiveness of such interventions, as they provide insights into the patient’s perspective and overall functionality. As the data originate exclusively from Germany, the findings may not necessarily generalize to countries or healthcare systems with different policies, resources, organizational barriers, or even patient demographics. This limits the wider generalizability of the findings and suggests a need for more comparative studies in diverse international settings. Furthermore, the absence of detailed data on the cost of treatment limits the practical utility of the findings. Detailed cost analyses are needed in future studies to inform healthcare policy and decision-making.

## 5. Conclusions

This study revealed a significant national increase in the use of outpatient hand prosthetic JR over the past decade. Compared to other large datasets, complication rates appear to have declined over the years, likely reflecting general improvements in the quality of the latest versions of hand joint implants. However, in contrast to international standards, inpatient settings still remain the preferred choice for these procedures among German providers. Nonetheless, there is a growing national trend towards outpatient JR procedures, notably without a significant increase in the rate of surgical revisions.

However, we are witnessing the beginning of a new era in hand JR therapy, which likely resembles the dynamics of JR therapies observed in large joints several decades ago. Looking ahead, cost-reducing initiatives will inevitably involve the management of these procedures in the outpatient setting. The system is still lagging behind in this regard, although data from this study, consistent with other reported outcomes, do not suggest any significant decline in quality for outpatient treatments.

## Figures and Tables

**Table 1:** The table lists OPS codes and their corresponding medical procedures.

OPS-Codes	OPS Group
5-824.a	Thumb carpometacarpal joint prosthesis
5-824.9	Finger joint prosthesis
5-824.90	1 finger joint prosthesis
5-824.91	2 finger joint prostheses
5-824.92	3 finger joint prostheses
5-824.93	4 finger joint prostheses
5-825.c	Removal of a finger joint prosthesis
5-825.d	Replacement of a thumb carpometacarpal joint prosthesis
5-825.e	Removal of a thumb carpometacarpal joint prosthesis
5-825.6	Replacement of a finger joint prosthesis

**Table 2:** Overview of the total number (n) of all patients who received a CMC or finger joint prosthesis, or who underwent a prosthesis-related revision surgery during the observed period. Absolute numbers and row percentages are shown. The differences between inpatient and outpatient sector were significant (chi-squared test) for all procedures.

		Total n	Outpatient (%)	Inpatient (%)	*p*
Number of procedures		22,752	3715 (16.3)	19,037 (83.7)	<0.001
Prosthesis implantation	All	19,501	3337 (17.1)	16,164 (82.8)	<0.001
	CMC-1	8782	1241 (14.1)	7541 (85.7)	<0.001
	Finger	10,719	2096 (19.6)	8623 (80.4)	<0.001
Revision surgeries		3251	378 (11.6)	2873 (88.4)	<0.001

**Table 3:** The incidence rate per 100,000 inhabitants (IR) for the years 2013 and 2022 are presented. Additionally, the incidence rate ratio (IRR) and the corresponding confidence interval are highlighted for in- and outpatient procedures. The significance levels were tested using the chi-squared test.

All		IR		IRR	95% CI	*p*
Year		2013	2022			
Number of procedures		2.56	3.99	1.56	1.47 to 1.64	<0.001
Prostheses	All	2.08	3.61	1.74	1.64 to 1.85	<0.001
	CMC-1	0.69	2.37	3.42	3.12 to 3.76	<0.001
	Finger	1.39	1.24	0.90	0.82 to 0.98	0.0111
Revisions		0.49	0.38	0.77	0.67 to 0.90	<0.001
**Outpatient**		**IR**		**IRR**	**95% CI**	***p***
Year		2013	2022			
Number of procedures		0.38	0.79	2.09	1.83 to 2.40	<0.001
Prostheses	All	0.34	0.74	2.18	1.89 to 2.51	<0.001
	CMC-1	0.12	0.40	3.44	2.73 to 4.33	<0.001
	Finger	0.22	0.34	1.53	1.27 to 1.84	<0.001
Revisions		0.04	0.05	1.36	0.86 to 2.15	0.1892
**Inpatient**		**IR**		**IRR**	**95% CI**	***p***
Year		2013	2022			
Number of procedures		2.19	3.20	1.46	1.38 to 1.55	<0.001
Protheses	All	1.74	2.88	1.65	1.55 to 1.77	<0.001
	CMC-1	0.58	1.98	3.42	3.08 to 3.79	<0.001
	Finger	1.16	0.90	0.77	0.70 to 0.85	<0.001
Revisions		0.45	0.32	0.72	0.62 to 0.85	<0.001

**Table 4:** The incidence rate for finger prostheses per 100,000 inhabitants (IR) ^1^ and the age-adjusted incidence rate (AAIR) for the years 2013 and 2022 are presented including incidence rate ratio (IRR) and the corresponding confidence interval. The significance level was tested using the chi-squared test.

All		IR ^1^ and AAIR		IRR	95% CI	*p*
		2013	2022			
Finger prostheses	All	1.39 ^1^	1.24 ^1^	0.90	0.82 to 0.98	0.0111
	40–59	1.18	1.15	0.98	0.83 to 1.15	0.7853
	60–79	4.35	3.50	0.81	0.73 to 0.89	<0.001
**Outpatient**		**IR ^1^ and AAIR**		**IRR**	**95% CI**	***p***
		2013	2022			
Finger prostheses	All	0.22 ^1^	0.34 ^1^	1.53	1.27 to 1.84	<0.001
	40–59	0.16	0.29	1.82	1.23 to 2.71	0.0026
	60–79	0.75	1.01	1.35	1.08 to 1.69	0.0076
**Inpatient**		**IR ^1^ and AAIR**		**IRR**	**95% CI**	***p***
		2013	2022			
Finger prostheses	All	1.16 ^1^	0.90 ^1^	0.77	0.70 to 0.85	<0.001
	40–59	1.02	0.87	0.85	0.70 to 1.02	0.0791
	60–79	3.60	2.49	0.69	0.61 to 0.78	<0.001

## Data Availability

Derived data supporting the findings of this study are available from the corresponding author upon reasonable request.
